# A 10-Week Block of Combined High-Intensity Endurance and Strength Training Produced Similar Changes in Dynamic Strength, Body Composition, and Serum Hormones in Women and Men

**DOI:** 10.3389/fspor.2020.581305

**Published:** 2020-09-30

**Authors:** Ritva S. Taipale, Jaakko Forssell, Johanna K. Ihalainen, Heikki Kyröläinen, Keijo Häkkinen

**Affiliations:** ^1^Sports Technology Unit, Faculty of Sport and Health Sciences, University of Jyväskylä, Vuokatti, Finland; ^2^Neuromuscular Research Center, Biology of Physical Activity, Faculty of Sport and Health Sciences, University of Jyväskylä, Jyväskylä, Finland

**Keywords:** sex differences, women, men, combined training, strength training, endurance training, high intensity training, hormones

## Abstract

**Purpose:** To examine the potential sex differences in adaptations to combined endurance and strength training in recreationally endurance trained (eumenorrheic) women (*n* = 9) and men (*n* = 10).

**Methods:** Isometric (ISOM_max_) and dynamic bilateral leg press (1RM), countermovement jump (CMJ), running performance (3,000 m time trial), lean mass and body fat % (LEAN and FAT% determined by dual X-ray absorptiometry) as well as serum testosterone and cortisol (TES and COR, respectively, measured using hormone-specific immunoassay kits) were examined before a control period and pre, mid, and post a supervised 10-week combined high-intensity interval endurance training (4 × 4 min intervals and 3 × 3 × 100 m repeated sprints) and mixed maximal and explosive strength training. No more than 2 weeks separated training and testing for either women or men and all women were tested in the early follicular phase of the menstrual cycle to minimize the possible influence of menstrual cycle phase on performance measures.

**Results:** Absolute and relative changes in 1RM, CMJ, 3,000 m, LEAN, and FAT% were similar between groups. The only statistically significant differences observed between groups were observed at post and included a larger Δ% increase in ISOM_max_ force in men and a relatively greater Δ% decrease in serum TES in women.

**Conclusion:** Women and men can achieve similar relative adaptations in dynamic maximal strength and CMJ as well as endurance performance gains and body composition over the same high-intensity 10-week combined program, although relative adaptations in TES may differ.

## Introduction

Differences in the anatomy and physiology of women and men result in a performance advantage for men of 8–12%, depending on sport discipline (Sandbakk et al., 2018). Women are generally smaller than men in terms of height, body mass, and muscle mass, while tending to have greater fat mass. Men generally have greater muscle force production characteristics than women that have partially been attributed to a greater proportional area of type II muscle fibers (Staron et al., 2000), however, these differences are attenuated when force production characteristics are normalized for lean mass although greater sex-differences persist in the upper body in comparison to the lower body (Sandbakk et al., 2018) indicating that sex differences in performance are strongly associated with lean mass. In contrast, in terms of aerobic capacity, men maintain a 5–10% performance advantage over women even relative to lean mass. The lower aerobic capacity in women is attributed to a smaller heart and lungs (Molgat-Seon et al., 2018) in addition to lower hemoglobin and oxygen transport capacity (Cureton et al., 1986; Bouwsema et al., 2017). While these anatomical and physiological differences exist between women and men, relative adaptations to strength training interventions in women and men appear to be similar (Knowles et al., 2019; Roberts et al., 2020). In contrast, men appear to respond and adapt to a given dose of endurance training to a greater extent than women (Diaz-Canestro and Montero, 2019) although endurance training interventions of moderate duration, e.g., 7 weeks, appear to induce similar adaptations in VO_2peak_ in both sexes (Carter et al., 2001). Interestingly, research comparing adaptations to combined endurance and strength training between women and men reveal contradictory results. Bell et al. (1997) reported that trained women may be more susceptible to interference than men, while Barnes et al. (2013) reported that female runners may benefit from heavy strength training more than men to improve measures of running performance. Taipale et al. (2014) reports that over 16 weeks of combined training women made greater improvements in maximal strength but men made more systematic improvements in submaximal running characteristics (Taipale et al., 2014).

Of the small number of studies examining differences in adaptations to combined training between women and men, few have taken the menstrual cycle into consideration aside from avoiding ovulation for hormonal analysis (e.g., Bell et al., 1997; Horne et al., 1997). As the menstrual cycle is a potential confounding factor when investigating training responses and adaptations in women taking menstrual cycle phase into consideration when comparing training adaptations between sexes may reveal sex differences that are otherwise “masked” by testing each women in random phase of their menstrual cycle. Research indicates that neuromuscular function (Petrofsky et al., 1976; Sarwar et al., 1996; Ansdell et al., 2019), absolute and relative VO_2max_ (Lebrun et al., 1995), and even the magnitude of adaptability to strength training (Reis et al., 1995; Sung et al., 2014; Wikström-Frisén et al., 2017) may be influenced by menstrual cycle phase while metabolism, body temperature (Janse DE Jonge et al., 2012) and even recovery (Hackney et al., 2019) may also fluctuate along with hormones. Indeed, recent meta-analyses indicate that the e.g., at least a small performance difference exists between menstrual cycle phases (McNulty et al., 2020) while hormonal contraceptive use may result in slightly inferior exercise performance (Elliott-Sale et al., 2020) although higher-quality research is still needed to confirm these observations.

The purpose of this study was to examine adaptations to 10 weeks of (different day) combined high-intensity endurance and mixed maximal and explosive strength training in women (eumenorrheic, not using hormonal contraceptives) and men, such that testing for women was completed in the early follicular phase to minimize the potential influence of menstrual cycle related hormonal fluctuation on performance measures. This is the first study examining the potential sex-differences in adaptations to combined endurance and strength training while taking into consideration and reporting menstrual cycle phase while clearly not mixing hormonal contraceptive users into the participant population. This approach is novel in that we investigate sex-differences while mitigating confounding factors related to both endogenous and exogenous hormones that may be “masking” meaningful sex differences.

## Materials and Methods

### Participants

Healthy, non-smoking, and recreationally endurance trained participants aged 18–40 were recruited. Inclusion criteria: BMI <30 kg/m^2^ and a 12 min running test result ≥2,300 m. Exclusion criteria: any diseases, musculoskeletal or cardiac problems, or medications (including hormonal contraceptives) that would preclude a participant's ability to perform the present training and testing. Participants filled-out a Finnish Physical Activity Readiness Questionnaire and responded to questions regarding their physical activity background prior to participation, next a resting ECG and health questionnaire from each participant were screened by a medical doctor. Verbal and written information about the measurement procedures, study design, and training were provided prior to the signing of an informed consent. Ethical approval was granted in August of 2014 by the University Ethical Committee. Ultimately, nine healthy women who had never used hormonal contraceptives and 10 healthy men completed the investigation. All included women reported regular menstrual cycles (24–35 days). Participant characteristics were measured at control (see Results section, **Table 2**).

### Study Design

Participants acted as their own controls over a 2–3 month period before the 10 week high-intensity combined endurance and strength training intervention during which they were instructed to continue with habitual physical activity, (primarily of low intensity aerobic exercise). As the aim of the study is to compare adaptations between women and men and a control period is reported, non-training groups of women and men in separate control groups were not included in the investigation. Women were tested during the early follicular phase of the menstrual cycle between days 1 5 counted from the commencement of menstrual bleeding and hormonally confirmed (Myllyaho et al., 2018). The intervention was planned such that no more than 2 weeks of “rest” (i.e., training outside of the intervention) separated training and testing for either women or men. Testing were completed at the beginning of the control period (control, included familiarization with all of the described testing methods) and at the end of the control period prior to the commencement of the 10-week training intervention (pre), half-way through the intervention (mid), and at the end of the intervention (post). Testing included anthropometry, measurement of force production and endurance capacity, as well as collection of blood samples for determination of resting levels of serum hormones. Testing was always completed at the same time of day (±2 hours) for each participant to control for circadian variations. Force production measurements were always completed prior to endurance measurements. The 10-week intervention was carried out in the autumn (August-November). Participants were asked continue with their established nutritional and hydration habits. The present data is a subset from a larger study (Myllyaho et al., 2018; Ihalainen et al., 2019; Taipale et al., 2019).

### Training

During the 10-week training intervention, a total of four high-intensity training sessions per week were performed on different days, by each participant, for a total of 37 training sessions. Training consisted of two strength training and two repeated interval running sessions per week. Improving force-production via periodized combined endurance and strength training has been shown to benefit running performance (Paavolainen et al., 1999; Støren et al., 2008; Barnes et al., 2013; Taipale et al., 2014) particularly when strength training exercises mimic the specific recruitment patterns of the endurance sport in question (Laursen et al., 2005) thus, strength training (Table 1) targeted the lower extremities but also included exercises for the core and upper body. Each strength training session included several multi-joint movements with maximal loads progressively increasing from 50 to 85% 1 RM as well as explosive/plyometric movements. This “mixed” training approach was aimed at improving both force and velocity components of force production (Newton and Kraemer, 1994). The main exercises were always performed first and included maximal and explosive sets of squat (performed with a barbell and weights and spotted by an experienced trainer), bilateral leg press starting at a knee angle of ~60 degrees (David 210, David Health Solution Ltd., Helsinki, Finland), knee flexion starting at a knee angle of ~100 degrees (David 200, David Health Solution Ltd., Helsinki, Finland), weighted calf raises, and calf jumps. These exercises were followed by explosive/plyometric sets of exercises without external load: bounding/strides, bench step-ups, and hurdle-jumps in which participants were instructed to do with maximal effort. Explosive exercises were divided into “A” and “B” programs that were performed once per week (Table 2). Exercise order was not specified for the main exercises, however, contrast movements were always performed after the heavier movement and the main exercises were always completed before explosive “A” and “B” programs while core and upper body were always trained at the end of the session. Strength training sessions were performed in a gym built for research purposes and supervised/documented by members of research staff.

**Table 1:** Strength training program.

	**Sets**	**Repetitions**	**Load** **(% 1RM)**	**Rest** **(min)**
**Main exercises (2x/week)**				
Back squat (barbell)	*1–2 warm-up*	8–10	50–70	1
	2 maximal	5–6	70–80 → 75–85	2
Squat jumps	2 explosive	10	Body weight	2
Leg press	*1–2 warm-up*	8–10	50–70	1
	2 maximal	5–6	70–80 → 80–85	2
	2 explosive	10	30–40	2
Knee flexion	*1–2 warm-up*	8–10	50–60	1
	2	8–10	70–80 → 75–85	2
Calf raises	*1 warm-up*	8–10	50–70	1
	2 maximal	5–6	70–80 → 75–85	2
Calf jump	2 explosive	6	Body weight	2
**Plyometrics A (once per week)**			
Bounding*(Alternating legs)*	2 explosive	6+6	Body weight	2
Hurdle jumps *(Serial bilateral jumps with high knees)*	2 explosive	6	Body weight	2
**Plyometrics B (once per week)**				
Bench step-ups *(Alternating legs)*	2 explosive	6+6	Body weight	2
Hurdle jumps *(Serial bilateral long jump)*.	2 explosive	6	Body weight	2
**Core and upper body (2x/week)**				
Bench press (barbell)	2–4	8–10	70–80	1–2
Back extension	2	10–15	0–5–10kg	1
Trunk rotation (obliques)	2	10–15	+10–20kg	1
Plank	2	60 s	Body weight	1

*The main exercises were performed 2x/week followed by either Plyometrics A or Plyometrics B. At the end of each session, the core and upper body were also trained*.

**Table 2:** Participant characteristics at pre-measurements.

**Group**	**n**	**Age** **(years)**	**Height (cm)**	**Body mass** **(kg)**	**Body fat (%)**	**VO_**2max**_** **(L·min^**−1**^)**	**VO_2max_ (ml·kg^−1^·min^−1^)**	**1RM** **(kg)**	**ISOM_**max**_ (N)**	**CMJ** **(cm)**
Men	10	32.6 ± 4.9	181.8 ± 4.6^¤¤¤^	79.3 ± 8.5^¤¤¤^	17.5 ± 5.2^¤^	3.8 ± 0.4 ^¤¤¤^	48.4 ± 4.1 ^¤^	164 ±27^¤¤¤^	3739 ± 1017^¤¤^	35.3 ± 4.8^¤¤^
Women	9	31.3 ± 5.4	168.3 ± 5.0	60.6 ± 9.2	23.9 ± 3.7	2.7 ± 0.3	43.6 ± 4.1	118 ± 18	2540 ± 684	26.2 ± 3.9
Difference women vs. men		4.0%	8%	31.0%	26.6%	40.5%	10.9%	39.5%	47.2 %	34.8%
Effect size women vs. men		*d* = 0.25 small	*d* = 2.81 large	*d* = 2.11 large	*d* = 1.42 large	*d* = 3.11 large	*d* = 1.17 large	*d* = 2.00 large	*d* = 1.38 large	*d* = 2.08 large

Significant difference between men and women:

¤ *p < 0.05*,

¤¤ *p < 0.01*,

¤¤¤ *p < 0.001*.

Endurance training sessions included two kinds of high-intensity interval (HIIT) training. One high-intensity 4 × 4 min running interval training session progressing in intensity from 70% maximal heart rate (HR_max_) to 90% of HR_max_ by the end of the training period [4 min active recovery at 60–70% HR_max_, similar to (Helgerud et al., 2007)], and one repeated sprint training session including three sets, of three repetitions of 100 m all-out sprints (2 min active recovery with 60–70% HR_max_ between repetitions and 5 min active recovery between sets) per week. Active recovery for our participants included running/brisk walking. Heart rate (HR) values were recorded to monitor training intensity. HR_max_ was determined using an incremental running test to exhaustion on a treadmill (see Methods).

Each week participants completed endurance and strength training sessions in the order in which they preferred, but the same training modality was not allowed on back-to-back days and a rest day was always required after 2 consecutive days of training in an effort to ensure adequate recovery. The order of exercise (strength or endurance first) does not appear to significantly affect training outcomes in all investigations (Eddens et al., 2018), however, training endurance and strength on separate days may be more effective than same-day combined training to improve endurance capacity and dynamic force production in both women and men (Eklund et al., 2016a). The participants were recreationally trained (familiar with strenuous exercise) and training was progressive, fatigue from training on back-to-back days was not expected to significantly affect training quality. Participants were encouraged to continue their other habitual physical activities during the study to ensure that their overall physical activity and training volume did not decrease. All training sessions were completed at approximately the same time of day.

### Testing

Participants warmed up at in individual intensity for 5 min with a cycle-ergometer prior to force measurements. The following measurements are reliable with an intra-class correlation in our laboratory of >0.7 as in Schumann et al. (2014) and participants were always verbally encouraged to achieve their best performance.

#### Maximal Bilateral Isometric Leg Press (ISOM_max_)

Isometric force production of the leg extensors was measured using an isometric horizontal bilateral leg press (Neuromuscular Research Center, Faculty of Sport and Health Sciences, University of Jyväskylä, Finland). Participant knee angle was 107 degrees determined from the greater trochanter, lateral tibiofemoral joint space, and lateral malleolus. Participants were instructed to produce as much force as possible as fast as they could for ~3 seconds. Force data was collected at a sampling frequency of 2,000 Hz, and then filtered (20 Hz low pass filter) and analyzed using customized scripts (Signal 4.10, CED, UK). A minimum of three trials (Häkkinen et al., 1998) was performed. If the maximum force during the last trial was >5% compared to the previous trial, an additional trial was performed up to a maximum of five trials. The best performance, in terms of maximal force, was used for statistical analysis.

#### Maximal Bilateral Leg Press (1RM)

Dynamic concentric force production of the leg extensor muscles was measured using a seated leg press device (David Sports Ltd., Helsinki, Finland). Participant knee angle at start was approximately 60 degrees (similar to training). Prior to attempting 1RM, participants warmed up as follows: 5x ~70%1RM, 3x ~80%1RM, and 2x ~90%1RM, with 1 min rests between sets. Following warm-up, participants were instructed to grasp handles located under the seat of the leg press and to keep constant contact with the seat and backrest during leg extension to a full range of motion (180 degrees). 1RM attempts were separated by a minimum rest of 1 min. Load was increased incrementally ~1.25–10 kg until the individual 1RM was found. No more than five attempts were performed. The greatest weight that a participant could successfully lift was recorded with an accuracy of 1.25 kg.

#### Countermovement Jump (CMJ)

To assess fast force production of the lower extremities, participants were instructed to jump as high as possible on a force platform (Neuromuscular Research Center, Faculty of Sport and Health Sciences, University of Jyväskylä, Finland) with an explosive countermovement action before the concentric phase of the movement. Participants performed three maximal CMJs with 1 min of rest in between. An additional trial was performed if the flight time from the third trial was 5% greater than the previous trial up to a maximum total of five trials. Force production and flight time were analyzed from all trials (Signal 4.10, CED, UK). Jump height was calculated manually from the force-time curve impulse using the equation h = I^2^·2 gm^−2^ (I = impulse, g = gravity, and m = mass of subject) (Kirby et al., 2011). The highest recorded jump height was included in statistical analyses.

#### Endurance Capacity

A 3,000 m time-trial was completed at control, pre, and post to assess potential changes in running performance during the intervention. After a 10 min individual running warm-up, participants ran the 3,000 m time-trial around an indoor 200 m athletics track. Split times and heart rate were recorded for each kilometer. A running test of this duration in a recreationally trained population correlates significantly with laboratory-determined oxygen-consumption data and this relationship makes it possible to estimate aerobic capacity with reasonable accuracy in field conditions (Cooper, 1968).

Maximal aerobic capacity was recorded using a standard protocol (Mikkola et al., 2007). Briefly, treadmill incline remained a constant 0.5 degrees while running velocity began at 7–9 km · h^−1^ and was increased by 1 km · h^−1^ every third min until volitional exhaustion. Heart rate was recorded continuously using a heart rate monitor (Suunto t6, Vantaa, Finland). Mean heart rate values from the last minute of each stage were used for analysis. Oxygen consumption was measured breath-by-breath using a portable gas analyzer (Oxycon Mobile®, Jaeger, Hoechberg, Germany) and VO_2max_ was accepted as the highest average 60 s VO_2_ value. Due to unanticipated treadmill laboratory maintenance, VO_2max_ data is presented only for control.

#### Body Composition

The height of each participant was measured using standard clinical methods prior to estimation of whole body composition using Dual X-ray Absorptiometry (LUNAR Prodigy, GE Medical Systems, Madison, WI, USA). DXA-scans were completed in the morning after a 12 h fast and visit to the bathroom. As with blood samples, participants were asked to refrain from strenuous physical activity for the 48 h prior to the measurements. Automatic analyses (Encore-software, version 14.10.022) provided total body fat percentage (FAT%) and total body lean mass.

#### Blood Samples

Blood samples were collected in conjunction with body composition measurements by a qualified lab technician into serum tubes (Venosafe Gel + Clot activator tubes, Terumo Medical Co., Belgium). The lab technician reviewed analyses of the basic blood count (collected in Venosafe EDTA Tubes, Terumo Medical Co., Belgium, and analyzed by Sysmex KX-21N, Kobe, Japan) for abnormalities that could indicate acute illness/infection. The samples were centrifuged for 10 min at a refrigerated temperature of +4°C with 2,000 × g (Heraeus Megafuge 1.0 R, Thermo Scientific, Karsruhe, Germany). Serum was kept at −80°C until analyzed for serum TES and COR. Samples were analyzed using hormone-specific immunoassay kits (Siemens, New York, NY, USA). Assay sensitivities were 0.5 nmol · L^−1^ and 5.5 nmol · L^−1^ for TES and COR, respectively. Inter-assay coefficients of variation were 9.4 and 6.6% for TES and COR, respectively.

#### Statistical Analysis

Statistics were performed using SPSS for Windows (IBM SPSS version 24.0; SPSS Inc., Chicago, IL). Means and standard deviations (SD) were calculated using conventional methods. The relative magnitude of changes (Δ%) were calculated as % change from control. All variables were analyzed using a two-way ANOVA with repeated measures (2–4 levels depending on the variable) and *post hoc* tests were performed with Bonferroni adjustments. Mauchly's test was used to test the assumption of sphericity. Where this assumption was violated, Greenhouse-Geisser adjustments were used. Where significant main effects or interactions were observed, pairwise comparisons were used to identify the location of differences between measurement time points and training. Statistical significance was set at *p* ≤ 0.05. Effect size (ES) was calculated using Cohen *d*. When *d* ≥ 0.2, ES was considered small; when *d* ≥ 0.5, ES was considered medium; and when *d* ≥ 0.8, ES was considered large (Cohen, 1988).

## Results

At control, height, body mass, body fat percentage, VO_2max_ (L·min^−1^ and ml·kg^−1^·min^−1^), and maximal bilateral dynamic strength (1RM) were statistically different between women and men (*p* < 0.05–*p* < 0.001). These differences were accompanied by a large ES (*d* ≥ 0.80), see Table 2.

Intervention training compliance was high with participants completing 96% of the 37 training sessions. No differences were observed in compliance to training between women and men or in compliance to endurance training vs. strength training.

### Force Production

**Figure 1:**
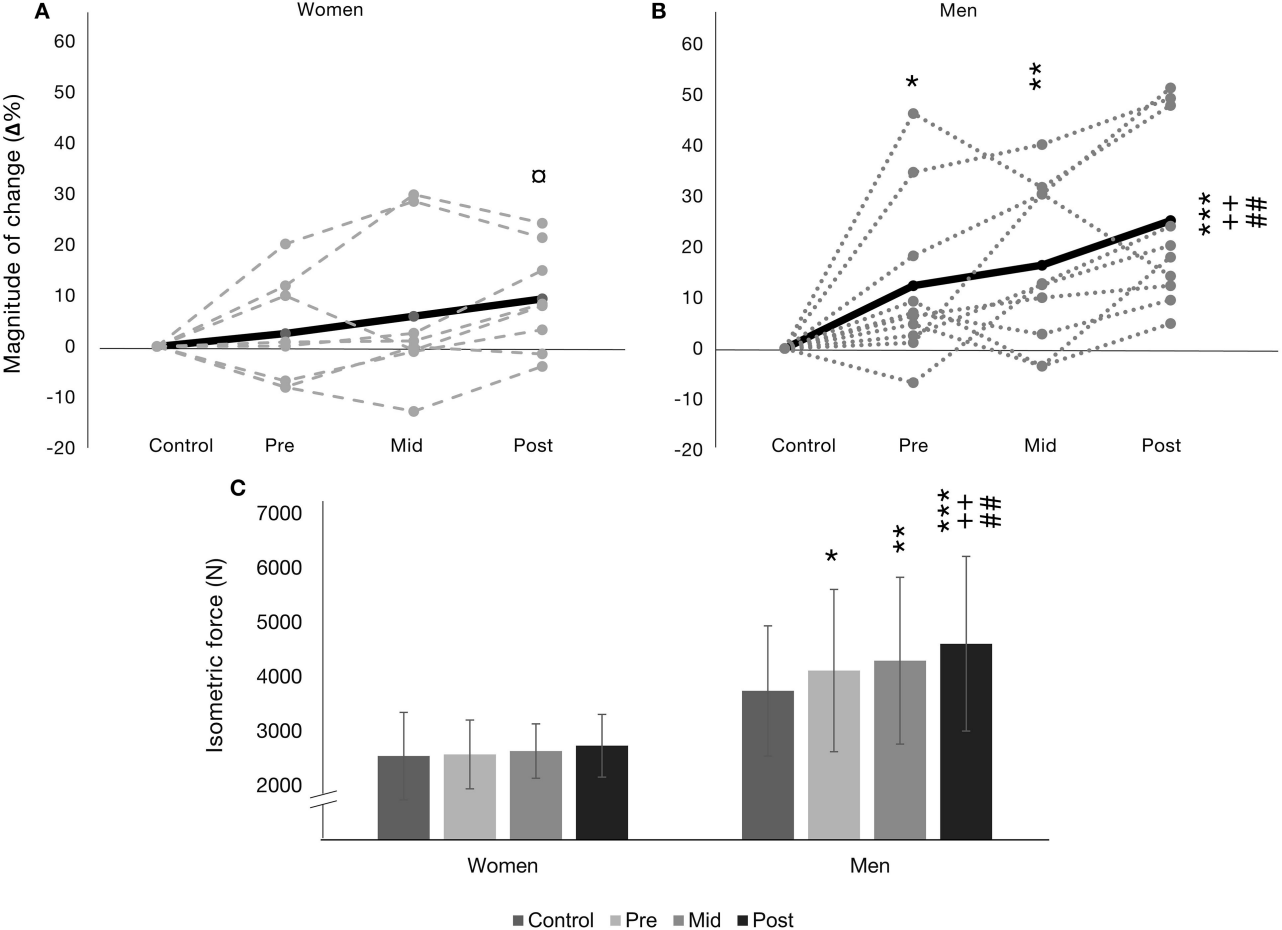
Isometric bilateral leg press (ISOM_max_) presented as relative magnitude of change (Δ%) in **(A)** for women (*n* = 8) and **(B)** for men (*n* = 10) as mean (black line) and individual changes (gray textured lines). **(C)** presents absolute means ± SD. *p < 0.05 from control, **p < 0.01 from control, ***p < 0.001 from control, ^++^*p* < 0.01 from pre, ^*##*^*p* < 0.01 from mid, and ^¤^*p* < 0.05 difference between women and men.

In terms of absolute ISOM_max_, a main effect for time (*F* = 9.98, *p* < 0.001) and group (*F* = 14.46, *p* < 0.01) were observed while a significant time × group interaction (*F* = 4.06, *p* < 0.05) was also observed (Figure 1C). No significant changes in absolute ISOM_max_ were observed in women from control to pre or from pre to post (2,571 ± 578–2,731 ± 559 N, *p* = n.s., *d* = 0. 28, small). Absolute ISOM_max_ increased progressively in men from control to pre (3,739 ± 1,071–4,114 ± 934 N, *p* < 0.05, *d* = 0.37, small) and pre to post (4,114 ± 934–4,606 ± 1,201 N, *p* < 0.01, *d* = 0.46, small). Absolute ISOM_max_ differed significantly between women and men (*p* < 0.05–*p* < 0.01, *d* = 1.33, 1.99, 2.49, 2.42, all large, at control, pre, mid, and post, respectively).

Figures 1A,B present Δ% ISOM_max_ for women and men, respectively, including indivdiual changes. A main effect for time (*F* = 11.32, *p* < 0.001) was observed, but no significant main effect for group or time × group interation was observed. A trend for Δ% ISOM_max_ increase in women was observed during the training intervention from pre to post (7 ± 6%, *p* = 059) while in men ISOM_max_increased significantly (12 ± 11 %, *p* < 0.01). A significant difference in Δ% ISOM_max_ was observed between men and women at post (*p* < 0.05, see Figure 1).

**Figure 2:**
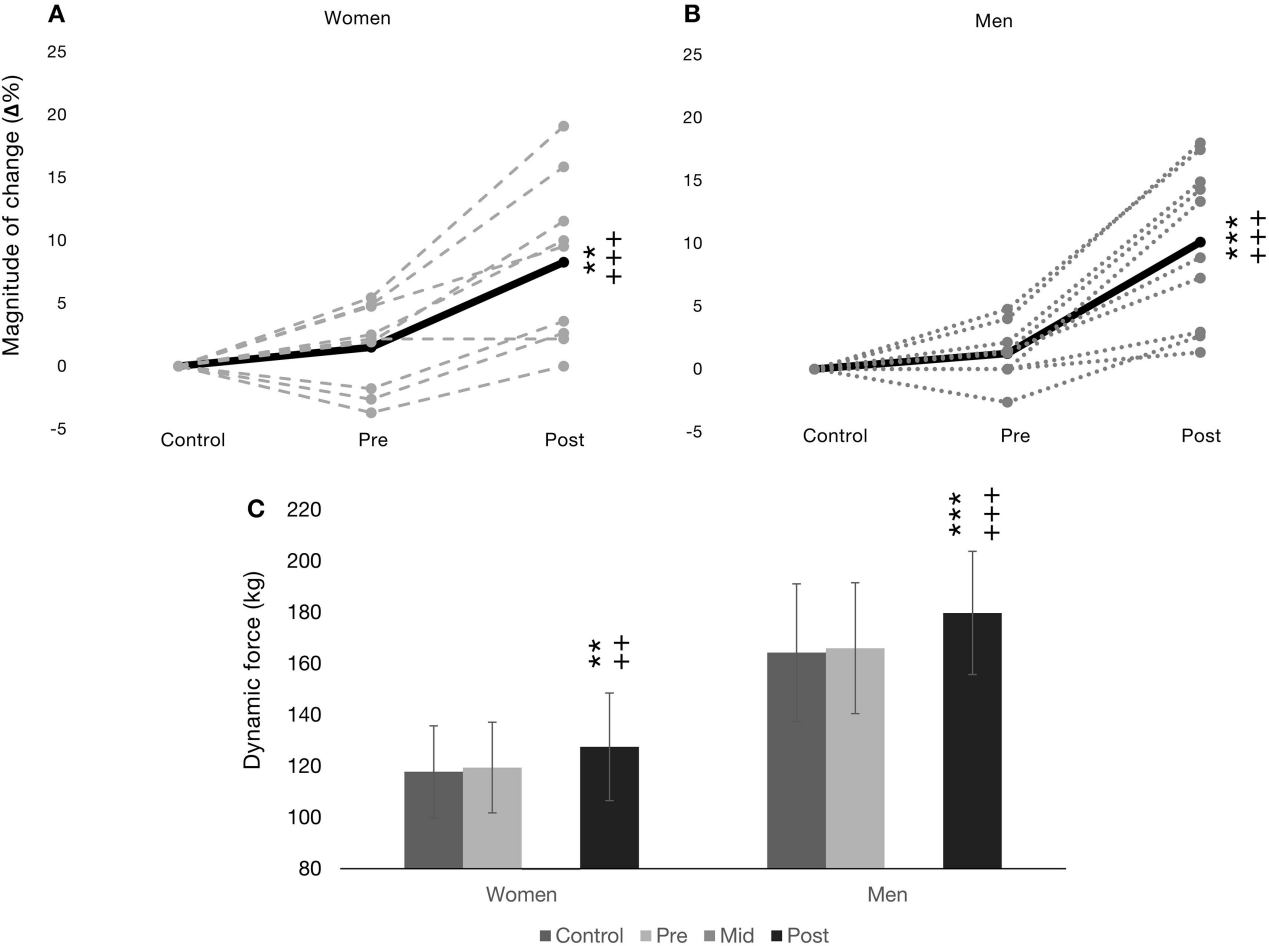
Dynamic bilateral leg press (1RM) presented as relative magnitude of change (Δ%) in **(A)** for women (*n* = 9) and **(B)** for men (*n* = 10) as mean (black line) and individual changes (gray textured lines). **(C)** presents absolute means ± SD. **p < 0.01 from control, ***p < 0.001 from control, ^++^*p* < 0.01 from pre, and ^+++^*p* < 0.001 from pre.

In terms of absolute dynamic 1RM, a significant main effect for time (*F* = 41.96, *p* < 0.001) and group were observed in (*F* = 22.24, *p* < 0.001), but a significant time × group interaction was not observed (Figure 2C). Significant increases in absolute 1RM were observed over the study period in both women and men from control to post as well as from pre to post (119 ± 18–128 ± 21 kg, *p* < 0.001, *d* = 0.46 small; 166 ± 26–180 ± 24 kg, *p* < 0.001, *d* = 0.56 medium, in women and men, respectively, see Figure 2). Absolute 1RM results between men and women were significantly different at all time points (*p* < 0.001, *d* = 2.20, 5.36, and 10.49, all large, at control, pre, and post, respectively).

Figures 2A,B present Δ% 1RM for women and men, respectively, including indivdiual changes. A significant main effect for time was observed in Δ% in 1RM (*F* = 39.82, *p* < 0.001), but no significant main effect was observed for group or time × group interaction. Significant increases in Δ% 1RM were observed over the training intervention in both women (7 ± 4%, *p* < 0.01) and men (9 ± 5%, *p* < 0.001) from pre to post, however, Δ% 1RM was similar between women and men at all time points.

**Figure 3:**
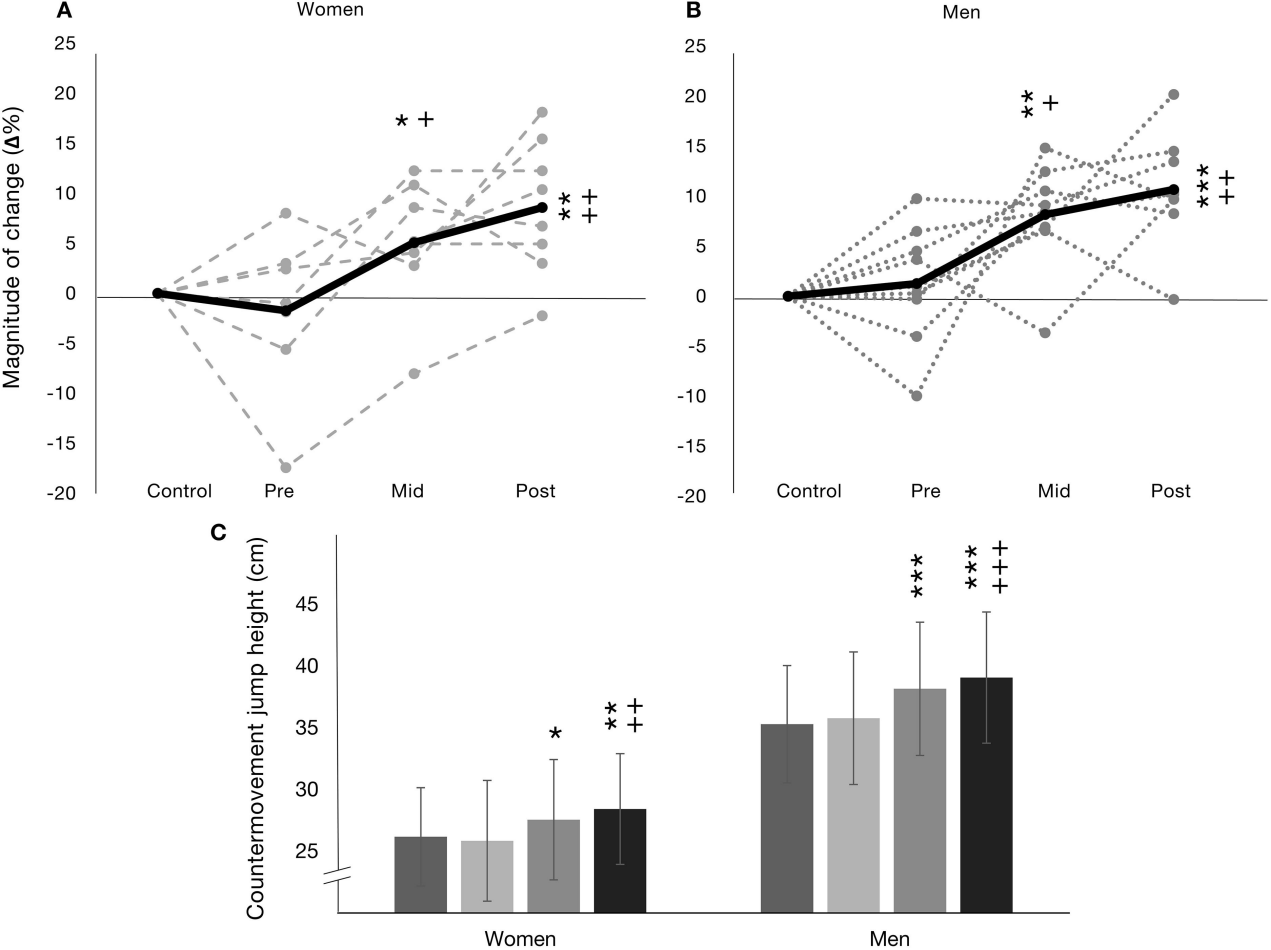
Countermovement jump (CMJ) presented as relative magnitude of changes (Δ%) in **(A)** for women (*n* = 8) and **(B)** for men (*n* = 9) as mean (black line) and individual changes (gray textured lines). **(C)** presents absolute means ± SD. *p < 0.05 from control, **p < 0.01 from control, ***p < 0.001 from control, ^++^*p* < 0.01 from pre, and ^+++^*p* < 0.001 from pre.

A significant main effects for time (*F* =16.59, *p* < 0.00) and group (*F* = 18.36, *p* < 0.001) were observed in absolute CMJ height but no time × group interaction was observed (Figure 3C). Both women and men significantly increased absolute jumping height during the training intervention (pre-post, 25.9 ± 5.1–28.4 ± 4.5 cm, *p* < 0.01, *d* = 0.53, medium; 35.8 ± 5.6–39.1 ± 5.6 cm, *p* < 0.001, *d* = 0.59, medium, for women and men, respectively). Absolute CMJ height between men and women was significantly different (*p* < 0.01, *d* = 2.09, 1.85, 2.06, and 2.11, all large, at control, pre, mid, and post, respectively).

Figures 3A,B present Δ% CMJ for women and men, respectively, including indivdiual changes. A main effect for time was observed in Δ% CMJ height (*F* = 17.85, *p* < 0.00) but not for group while no time × group interaction was observed. Significant increases in Δ% CMJ height were observed over the training intervention in both women (11 ± 6%, *p* < 0.01) and men (10 ± 8%, *p* < 0.001) from pre to post, however, Δ% CMJ height between women and men was similar at all time points.

No significant main effect for time or group was observed while no time × group interaction was observed in absolute 3,000 m time trial or Δ% 3,000 m time-trial times (Table 3). In addition, no sigificant differences between groups were observed at any time point in absolute or Δ% 3,000 m time-trial times. Control running times were 13:11 ± 0:31 (*n* = 5) and 12:53 ± 1:17 (*n* = 10) for women and men, respectively. Women improved their running time by 2.0 ± 1.9% (13:23 ± 1:25–13:09 ± 1:22, n.s., *d* = 0.17, small) between pre and post while men improved their running time by 2.3 ± 3.1% (13:07 ± 1:19–12:47 ± 1:02, *p* < 0.05, *d* = 0.27, small). The ES between groups ranged from *d* = 0.21–0.31, small at control, pre, and post.

**Table 3:** Mean ± SD serum testosterone and cortisol concentrations.

**Group**		**Men(*n* = 9)**	**Women (*n* = 5)**	**Effect sizewomen vs. men**
Testosterone (nmol · L^−1^)	Control	15.48 ± 3.63^¤¤¤^	0.79 ± 0.27	*d* = 5.7 large
	Pre	15.68 ± 5.01^¤¤¤^	0.59 ± 0.16	*d* = 4.6 large
	Mid	16.13 ± 3.47^¤¤¤^	0.75 ± 0.27	*d* = 6.2 large
	Post	15.46 ± 4.73^¤¤¤^	0.47 ± 0.15	*d* = 4.48 large
Effect size Pre-post		*d* = 0.05 small	*d* = 0.77 medium	-
Cortisol (nmol · L^−1^)	Control	451 ± 116	530 ± 102	*d* = 0.72 medium
	Pre	451 ± 126	545 ± 94	*d* = 0.85 large
	Mid	419 ± 81^¤^	567 ± 153	*d* =1.21 large
	Post	411 ±134	529 ± 243	*d* = 0.60 medium
Effect size Pre-post		*d* = 0.31 medium	*d* = 0.09 small	-

¤¤¤ *p < 0.001 = significant difference in absolute TES concentrations between women and men*.

¤ *p < 0.05 = significant difference in absolute COR concentrations between women and men*.

### Serum Hormones

No significant main effect for time or time × group interaction was observed in absolute TES concentrations, but a significant main effect for group (*F* = 75.44, *p* < 0.001) was observed (Table 3). Absolute concentrations of TES remained stable in both women and men while TES in men was significantly higher than in women at control, pre, mid, and post (*p* < 0.001, *d* ≥ 0.80, large, see Table 3).

In terms of Δ% TES, a significant main effect for time (*F* = 4.07, *p* < 0.05) and time × group interaction (*F* = 3.23, *p* < 0.05) were observed but no significant main effect for group was observed. From control to post and mid to post, Δ% TES was significant in women (*p* < 0.01 and *p* < 0.001, respectively, see Table 3). At post, a significant difference between women and men was observed with a greater Δ% TES decrease observed in women from control to post (*p* < 0.01).

For absolute COR, no significant main effect for time or group were observed and no significant time × group interaction was observed (Table 3). Absolute concentrations of COR remained stable in both women and men over the study period and only a transient difference in COR concentrations was observed between women and men at mid (*p* < 0.05).

In terms of Δ% COR concentrations, no significant main effects for time or group were observed while no significant time × group interaction was observed.

### Body Composition

Significant main effects for time and group were observed in absolute lean mass (*F* = 47.86, *p* < 0.001 and *F* = 76.19, *p* < 0.001, respectively) while a significant time × group interaction was also observed in absolute lean mass (*F* = 4.61, *p* < 0.05). Lean mass increased significantly from pre to post in both women (*n* = 9, 43.8 ± 2.6–44.7 ± 2.8 kg, 2.1 ± 1.0%, *p* < 0.01, *d* = 0.34, medium) and in men (*n* = 10, 62.4 ± 5.9–64.1 ± 6.2 kg, 2.8 ± 1.7%, *p* < 0.001, *d* = 0.29, small) while significant difference between women and men in terms of lean mass was observed at both pre and post (*p* < 0.001, *d* = 4.09 and 4.06, both large, respectively). Lean mass Δ% was similar between women and men from pre to post.

In the lower extremities, no significant main effect for time was observed, but a main effect for group was observed in absolute leg lean mass (*F* = 3.86, *p* = 0.066 and *F* = 78.1, *p* > 0.05, respectively) while no significant time × group interaction was observed (*F* = 2.77, *p* > 0.05). Leg lean mass increased in women (*n* = 9, 14.7 ± 1.0–15.1 ± 1.2 kg, 3.0 ± 2.1%, *p* > 0.05, *d* = 0.41, medium) and in men (*n* = 10, 21.5 ± 2.1–22.3 ± 2.5 kg, 4.1 ± 13.8%, *p* < 0.05, *d* = 0.37, medium) while a significant difference between women and men was observed at both pre and post (*p* < 0.001, *d* = 4.17 and 3.67, both large, respectively). A statistical trend (*p* = 0.051) indicated a slight difference in leg lean mass Δ% between women and men from pre to post.

Significant main effects for time and group were observed in FAT% (*F* = 11.26, *p* < 0.01 and *F* = 5.66, *p* < 0.05), but no time × group interaction was observed. A significant decrease in FAT% was observed in women (*n* = 9, 23.9 ± 0.1–22.4 ± 0.1 kg, *p* < 0.01, *d* = 0.24, small), but no significant change was observed in men (*n* = 10, 17.5 ± 5.2–16.7 ± 4.2 kg, *p* > 0.05, *d* = 0.17, very small). Signficant differences between women and men were observed at pre (*p* < 0.05, *d* = 1.06, large) and post (*p* < 0.05, *d* = 1.10, large). FAT% Δ% were similar between women and men from pre to post.

## Discussion

The present 10-week intervention was successful in inducing significant improvements in 1RM and CMJ in both women and men, but only modest gains in aerobic 3,000 m running time were observed in either group. Changes in absolute and relative (Δ%) performance measures were similar in women and men except for ISOM_max_, which did not increase significantly over the control period or training intervention in women. Progressive and significant improvements in ISOM_max_ were, however, observed over both the control period and training intervention in men culminating in a significant difference between women and men in Δ% ISOM_max_ at post. The 10-week training intervention also induced positive changes in body composition including significant increases in lean mass in both women and men whereas only women achieved a significant decrease in FAT%. Ultimately, however, Δ% in body composition changes were similar between women and men. In terms of morning serum hormonal concentrations, a relatively higher magnitude of decrease in TES was observed in women when compared to men at post although absolute concentrations remained unaltered throughout the control period and training intervention with concentrations remaining well within clinical norms in both women and men.

Women and men in the present study were matched for age and physical activity background, however, men had a statistically higher level of aerobic fitness (VO_2max_ in L· min^−1^ and ml · kg^−1^ · min^−1^), absolute maximal (ISOM_max_ and 1RM) and fast-force production (CMJ), body mass, and lean mass in addition to lower FAT% that were present throughout the control period and training intervention and accompanied by large ES. The differences in control values for force production and aerobic capacity are explained, at least in part, by the sex differences presented in the introduction and are in line with the sex differences of ~25–40% in maximal lower body strength and ~45–55% difference in aerobic capacity (VO_2max_ in L · min^−1^) observed between similarly trained women and men (Sandbakk et al., 2018). The slightly larger difference in strength and aerobic capacity between women and men in the present study might be attributed to our participants only being recreationally trained as training appears to mitigate performance differences between sexes (Sandbakk et al., 2018). We recognize that similarly trained women and men may not always be stratified into the same fitness categories, however, the present study population is considered to have “excellent” aerobic capacity by the American College of Sports Medicine (Liguori et al., 2014) while running performance was also similar as illustrated by small ES. As such, we feel comfortable in comparing training adaptations between the present groups of women and men.

Dynamic force production assessed by 1RM and CMJ remained unchanged during the control period, as expected, for both women and men, whereas both groups significantly improved 1RM and CMJ, however, with only small to medium ES, over the training intervention. The Δ% 1RM and Δ% CMJ were similar between women and men although the significant absolute differences observed between women and men in 1RM and CMJ at control were, naturally, maintained. Increases in maximal force production coincide with increases in explosive or fast-force production capabilities (Newton and Kraemer, 1994) as both require activation of type II muscle fibers. The observed increases in 1RM are somewhat smaller than those reported in other combined endurance and strength training studies including women and men. Bell et al. (1997) reported ~10% increase in dynamic incline leg press over 10 weeks in women and ~20% increase in men while Eklund et al. (2016b) reported ~16% increase in dynamic leg press over 12 weeks in women and ~10% increase in men. Endurance training may blunt adaptations to strength training (Hunter et al., 1987; Leveritt et al., 1999) a phenomenon known as the “interference effect” (Hickson, 1980). In particular, improvements in maximal force production appear to be compromised when combined training volume is high (Hickson, 1980) while explosive (fast) force production may be blunted when combined training duration is long (Häkkinen et al., 2003). In the present study, HIIT endurance training combined with strength training may be responsible for the smaller gains in lower body force production capabilities in line with (Sabag et al., 2018). The relatively small increase in strength might also be related to endurance training mode as running requires repetitive low-moderate force production that utilizes the stretch-shortening cycle (Komi, 2000), which is markedly faster and shorter than what is required in cycling or rowing and is anticipated to interfere more with maximal force production and muscle hypertrophy (Wilson et al., 2012). On the other hand, strength development during low volume combined training (2x per week over 11 weeks) can be similar regardless of type or intensity of aerobic training (Silva et al., 2012).

In women, no changes in absolute ISOM_max_ were observed during the control period or the investigation while significant improvements in absolute ISOM_max_ were observed during both the control period, and training intervention in men. Significant differences is absolute ISOM_max_ between women and men were maintained throughout the investigation while Δ% ISOM_max_ was significantly different between women and men at post. While a relationship exists between isometric and dynamic force production and improvements in ISOM_max_ might be expected in both women and men, changes in ISOM_max_ and 1RM after strength training are not necessarily related and different mechanisms may be responsible for adaptations (Baker, 1994). Semmler and Enoka (2000) explain that isometric force-production is strongly linked to muscle size and somewhat less to muscle activation whereas dynamic movements are reliant on muscle size as well as muscle activation/coordination (Semmler and Enoka, 2000). It is therefore possible that women and men both improved muscle activation/coordination during the present intervention, but that the slightly larger Δ% increase in lean mass of the lower extremities, and even more so the greater absolute muscle size in the lower extremities of men may, in part, explain this discrepancy. Unfortunately, we cannot determine the specific differences in time-course of adaptations in muscle activation and hypertrophy in the present study.

Small increases in fast-force production capabilities may be accompanied by improved endurance performance even without changes in VO_2max_ (Johnston et al., 1997; Paavolainen et al., 1999; Spurrs et al., 2003). In the present investigation, mean 3,000 m running times were similar between women and men, and accompanied by small ES. Mean 3,000 m running times did not change significantly during the control period indicating that the present training intervention did not provide an adequate stimulus to improve running performance while women and men responded similarly to the training stimulus. Although strength training is often credited with improving running performance by way of improved neuromuscular characteristics (Paavolainen et al., 1999; Taipale et al., 2010) improvements may not be evident over shorter running distances. In line with this investigation, a 10-week maximal strength training intervention in recreational runners (women) did not improve 3,000 m running performance despite significant improvements in force production (Kelly et al., 2008). In retrospect, the endurance training component of the present training intervention could have started at an even higher intensity and could have included a higher number of training sessions or interval repetitions in order to, potentially, elicit greater improvements in endurance performance. The volume of the HIIT training in the present intervention was less than half (1x per week) of that employed in Helgerud et al. (2007), in which high intensity intervals (3x per week over 8 weeks) in an endurance trained population achieved a significant 7% increase in aerobic capacity measured as L · min^−1^ (Helgerud et al., 2007). It is important, however, to consider that an increase in endurance training load may have also “interfered” with strength development (Hickson, 1980; Häkkinen et al., 2003) and that “interference” may present itself in different ways in women and men (Bell et al., 1997). It is worth noting that individuals respond to training in unique ways even when an effort is made to “match” groups as several variables contribute to individual responses to training (Mann et al., 2014; Ahtiainen et al., 2016; Pickering and Kiely, 2019). Progressive and closely supervised training might not elicit the same adaptation or same time-course of adaptations in all participants.

While the present training was not focused on improving body composition, women and men significantly increased whole body lean mass by 2–3% over 10 weeks while only women significantly decreased whole body FAT%. The magnitude of change in both lean mass and FAT% between women and men was, however, similar although ES indicate that the increase in lean mass in women was more meaningful that that in men (medium vs. small). The finding of increased lean mass in both women and men is in line with previous investigations using “different day” combined endurance and strength training. Eklund et al. (2016b) reported a slightly greater magnitude (3–4%) of increase in lean mass in both women and men over 12 weeks of “different day” combined training using circuit (2–4 sets of 15–20 reps at 60% 1RM) progressing to hypertrophic (2–5 sets ×8–12 reps at 80–85% 1RM) training and cycling performed at an intensity near aerobic threshold at the same training frequency as the present study. The slightly greater increase in lean mass in Eklund et al. (2016b) may be attributed to the endurance training mode of cycling that is generally recognized as a more favorable endurance stimulus to combine with strength training than running, when aiming to increase lower body strength and muscle mass (Wilson et al., 2012).

Absolute TES concentrations were lower in women than in men and these remained unaltered during the training intervention although medium and small ES accompanied the minor decreases in mean TES concentrations observed from pre to post in both women and men, respectively. The Δ% decrease of TES in women was significantly larger in women than in men. Interestingly, although COR concentrations were similar between women and men throughout the control period and intervention, medium to large ES were observed between groups indicating that difference in concentrations of COR between women and men are meaningful. The only statistically significant difference in absolute COR concentrations between women and men was transient and occurred at mid. COR concentrations were unaltered during the intervention although small and medium ES accompanied the minor decreases in mean COR concentrations observed from pre to post in both women and men, respectively. The Δ% changes in COR did not differ between women and men. The relatively low volume of training (4x/week) was not expected to cause significant changes in basal hormonal concentrations while the present combination of maximal and explosive strength training is unlikely to cause as much metabolic stress or hormonal response as e.g., hypertrophic training (Kraemer et al., 1993; Linnamo et al., 2005).

Previous comparisons of hormonal responses between women and men to a given combined endurance and strength training period indicate that women may have a greater increase in (urinary) COR in response to training while TES generally appears to remain stable (Horne et al., 1997; Bell et al., 2000). Other studies have demonstrated that both TES and COR concentrations may remain stable even over 16 weeks of combined endurance and strength training in both women and men (Taipale et al., 2014). In a 24 week training intervention Eklund et al. (2016a) demonstrated that training order in women may increase basal levels of COR, particularly when strength training is performed before endurance training in a single session, however, this increase was not statistically different from the group performing endurance training before strength training. More importantly, when women completed endurance and strength training on separate days, no changes in basal concentrations of TES or COR were observed (Eklund et al., 2016b).

One might anticipate that the high-intensity nature of the present combined training could have caused transient changes in absolute hormone concentrations; however, acute responses to training are unlikely to be observed in basal measurements. While the activity of the pituitary-adrenocortical system may be an indicator of training stress or training intensity (Brownlee et al., 2005; Tremblay et al., 2005) and while TES and COR (and their ratio) have been used to indicate overreaching/overtraining and of an anabolic/catabolic state (Häkkinen et al., 1985; Adlercreutz et al., 1986), we should recognize that the observed increase in COR may actually be a favorable adaptation (Viru and Viru, 2004) and that negative training stress may not be fully reflected in basal concentrations of the measured hormones (Kuoppasalmi et al., 1980; Consitt et al., 2002).

In the studies mentioned above, Bell et al. (1997), Horne et al. (1997), and Bell et al. (2000) state that study participants were not using hormonal contraceptives, and that ovulation ±3 days was avoided when taking blood samples (Bell et al., 1997, 2000; Horne et al., 1997), however, this means that performance measurements have been completed in both follicular and luteal phases, which, in eumenorrheic women are characterized by a different hormonal milieu. In the present investigation, we were successful in collecting performance data and blood samples when women were in the early follicular phase of their menstrual cycle when serum sex hormone concentrations are at their lowest (Myllyaho et al., 2018). While some research suggests that the menstrual cycle does not significantly influence hormonal responses to exercise (Galliven et al., 1997; O'Leary et al., 2013) contradictory reports also exist (Kraemer et al., 1995; Hornum et al., 1997; Kanaley et al., 2001; Nakamura et al., 2011). Likewise, the literature on the topic of menstrual cycle phase affecting endurance and/ or neuromuscular performance (Petrofsky et al., 1976; Quadagno et al., 1991; Sarwar et al., 1996; Elliott-Sale et al., 2003; Kubo et al., 2009; Ansdell et al., 2019) is equivocal. Nevertheless, by completing measurements for women in the early follicular phase we can minimize the potential influence of the hormonal spike associated with ovulation and higher concentrations of estrogen and progesterone that are observed in the luteal phase of the menstrual cycle phase. In addition, given that the magnitude of adaptability to strength training may be influenced by menstrual cycle phase (Reis et al., 1995; Sung et al., 2014; Wikström-Frisén et al., 2017) and that metabolism and body temperature (Janse DE Jonge et al., 2012) as well as recovery (Hackney et al., 2019) may fluctuate concomitant to hormonal fluctuations, we can postulate that any potential advantage or disadvantage of performing training in a certain phase of the cycle (Reis et al., 1995; Sung et al., 2014; Wikström-Frisén et al., 2017) are minimized although it is important to recognize that each woman's hormonal profile is unique and menstrual cycles are impossible to match.

## Limitations

For the present study we were only realistically able to test performance measures in women during a single phase of the menstrual cycle. As such, we tested women in the follicular phase of the menstrual cycle when serum sex hormone concentrations are at their lowest. Testing in the luteal phase when serum sex hormone concentrations are higher may have revealed different results. The sample size of the present investigation was relatively small although it is in line with other publications. We acknowledge these short-comings in our work, but want to emphasize the strengths of our study. All participants were highly motivated to take part in the study and we were able to control the above mentioned, possibly confounding factors related to endogenous and exogenous hormone concentrations. In addition, our research team took great care to continually interact with each participant during the supervised training sessions and throughout the study to ensure compliance with the study protocols and motivation was maintained.

## Conclusions

The present high-intensity combined endurance running and maximal and explosive strength training is appropriate for improving 1RM, CMJ, and body composition in a similar manner in women and men with similar training background and aerobic fitness when menstrual cycle phase is taken into consideration for testing. Minor improvements in 3,000 m running performance improvements are also similar between women and men while relative hormonal responses in terms of TES appear to differ. The present study indicates that women and men can complete high intensity combined endurance and strength training with the same periodization and programing, for at least 10 weeks, to achieve similar results. Dynamic measurements may be more appropriate for assessing strength development over a 10 week training block in women while different testing methods or a more potent endurance training stimulus may be necessary to observe improvements in running performance. Careful consideration of endurance training mode and strength training loads as well as their combined intensity is necessary while further investigation of potential sex differences is warranted.

## Data Availability Statement

The raw data supporting the conclusions of this article will be made available by the authors, without undue reservation.

## Ethics Statement

The studies involving human participants were reviewed and approved by University of Jyväskylä Ethical Committee. The participants provided their written informed consent to participate in this study.

## Author Contributions

RT, KH, and HK conceived and designed research. JF and JI conducted experiments. RT, JF, and JI analyzed data. All authors contributed to writing the manuscript. All authors have approved the manuscript.

## Conflict of Interest

The authors declare that the research was conducted in the absence of any commercial or financial relationships that could be construed as a potential conflict of interest.

## References

[B1] Adlercreutz H. Harkonen M. Kuoppasalmi K. Naveri H. Huhtaniemi I. Tikkanen H.. (1986). Effect of training on plasma anabolic and catabolic steroid hormones and their response during physical exercise. Int. J. Sports Med. 7, 27–28. 10.1055/s-2008-1025798 3744643

[B2] Ahtiainen J. P. Walker S. Peltonen H. Holviala J. Sillanpää E. Karavirta L.. (2016). Heterogeneity in resistance training-induced muscle strength and mass responses in men and women of different ages. Age 38, 1–13. 10.1007/s11357-015-9870-1 PMC5005877 26767377

[B3] Ansdell P. Brownstein C. G. Skarabot J. Hicks K. M. Simoes D. C. M. Thomas K.. (2019). Menstrual cycle-associated modulations in neuromuscular function and fatigability of the knee extensors in eumenorrheic women. J. Appl. Physiol. 126, 1701–1712. 10.1152/japplphysiol.01041.2018 30844334

[B4] Baker D. (1994). Generality versus specificity: a comparison of dynamic and isometric measures of strength and speed-strength. Artic. Eur. J. Appl. Physiol. Occup. Physiol. 68, 350–355. 10.1007/BF00571456 8055895

[B5] Barnes K. R. Hopkins W. G. Mcguigan M. R. Northuis M. E. Kilding A. E. (2013). Effects of resistance training on running economy and cross-country performance. Med. Sci. Sports Exerc. 45, 2322–2331. 10.1249/MSS.0b013e31829af603 23698241

[B6] Bell G. Syrotuik D. Socha T. Maclean I. Quinney H. A. (1997). Effect of strength training and concurrent strength and endurance training on strength, testosterone, and cortisol. J. Strength Cond. Res. 11, 57–64. 10.1519/00124278-199702000-00012

[B7] Bell G. J. Syrotuik D. Martin T. P. Burnham R. Quinney H. A. (2000). Effect of concurrent strength and endurance training on skeletal muscle properties and hormone concentrations in humans. Eur. J. Appl. Physiol. 81, 418–427. 10.1007/s004210050063 10751104

[B8] Bouwsema M. M. Tedjasaputra V. Stickland M. K. (2017). Are there sex differences in the capillary blood volume and diffusing capacity response to exercise? J. Appl. Physiol. 122, 460–469. 10.1152/japplphysiol.00389.2016 27932673 PMC5401957

[B9] Brownlee K. K. Moore A. W. Hackney A. C. (2005). Relationship between circulating cortisol and testosterone: influence of physical exercise. J. Sports Sci. Med. 4, 76–83. 24431964 PMC3880087

[B10] Carter S. L. Rennie C. D. Hamilton S. J. Tarnopolsky M. A. (2001). Changes in skeletal muscle in males and females following endurance training. Can. J. Physiol. Pharmacol. 79, 386–392. 10.1139/y01-008 11405241

[B11] Cohen J. (1988). Statistical Power Analysis for the Behavioral Sciences, 2nd Edn. Hilsdale, NJ: Lawrence Earlbaum Associates.

[B12] Consitt L. A. Copeland J. L. Tremblay M. S. (2002). Endogenous anabolic hormone responses to endurance versus resistance exercise and training in women. Sports Med. 32, 1–22. 10.2165/00007256-200232010-00001 11772159

[B13] Cooper K. H. (1968). A means of assessing maximal oxygen intake: correlation between field and treadmill testing. J. Am. Med. Assoc. 203, 201–204. 10.1001/jama.1968.03140030033008 5694044

[B14] Cureton K. Bishop P. Hutchinson P. Newland H. Vickery S. Zwiren L. (1986). Sex difference in maximal oxygen uptake - effect of equating haemoglobin concentration. Eur. J. Appl. Physiol. Occup. Physiol. 54, 656–660. 10.1007/BF00943356 3948861

[B15] Diaz-Canestro C. Montero D. (2019). Sex dimorphism of VO_2max_ trainability: a systematic review and meta-analysis. Sports Med. 49, 1949–1956. 10.1007/s40279-019-01180-z 31494865

[B16] Eddens L. van Someren K. Howatson G. (2018). The role of intra-session exercise sequence in the interference effect: a systematic review with meta-analysis. Sports Med. 48, 177–188. 10.1007/s40279-017-0784-1 28917030 PMC5752732

[B17] Eklund D. Häkkinen A. Laukkanen J. A. Balandzic M. Nyman K. Häkkinen K. (2016a). Fitness, body composition and blood lipids following 3 concurrent strength and endurance training modes. Appl. Physiol. Nutr. Metab. 41, 767–774. 10.1139/apnm-2015-0621 27351384

[B18] Eklund D. Schumann M. Kraemer W. J. Izquierdo M. Taipale R. S. Häkkinen K. (2016b). Acute endocrine and force responses and long-term adaptations to same-session combined strength and endurance training in women. J. Strength Cond. Res. 30, 164–175. 10.1519/JSC.0000000000001022 26020708

[B19] Elliott-Sale K. Cable N. T. Reilly T. Diver M. J. (2003). Effect of menstrual cycle phase on the concentration of bioavailable 17-β oestradiol and testosterone and muscle strength. Clin. Sci. 105, 663–669. 10.1042/CS20020360 12848619

[B20] Elliott-Sale K. J. McNulty K. L. Ansdell P. Goodall S. Hicks K. M. Thomas K.. (2020). The effects of oral contraceptives on exercise performance in women: a systematic review and meta-analysis. Sports Med. 10.1007/s40279-020-01317-5. [Epub ahead of print]. 32666247 PMC7497464

[B21] Galliven E. A. Singh A. Michelson D. Bina S. Gold P. W. Deuster P. A. (1997). Hormonal and metabolic responses to exercise across time of day and menstrual cycle phase. J. Appl. Physiol. 83, 1822–1831. 10.1152/jappl.1997.83.6.1822 9390951

[B22] Hackney A. C. Kallman A. L. Aggön E. (2019). Female sex hormones and the recovery from exercise: menstrual cycle phase affects responses. Biomed. Hum. Kinet. 11, 87–89. 10.2478/bhk-2019-0011 31179123 PMC6555618

[B23] Häkkinen K. Alén M. Kraemer W. J. Gorostiaga E. Izquierdo M. Rusko H.. (2003). Neuromuscular adaptations during concurrent strength and endurance training versus strength training. Eur. J. Appl. Physiol. 89, 42–52. 10.1007/s00421-002-0751-9 12627304

[B24] Häkkinen K. Kallinen M. Izquierdo M. Jokelainen K. Lassila H. Malkia E.. (1998). Changes in agonist-antagonist EMG, muscle CSA, and force during strength training in middle-aged and older people. J. Appl. Physiol. 84, 1341–1349. 10.1152/jappl.1998.84.4.1341 9516202

[B25] Häkkinen K. Pakarinen A. Alén M. Komi P. V. (1985). Serum hormones during prolonged training of neuromuscular performance. Eur. J. Appl. Physiol. Occup. Physiol. 53, 287–293. 10.1007/BF00422840 4039254

[B26] Helgerud J. Hoydal K. Wang E. Karlsen T. Berg P. Bjerkaas M.. (2007). Aerobic high-intensity intervals improve VO_2max_ more than moderate training. Med. Sci. Sports Exerc. 39, 665–671. 10.1249/mss.0b013e3180304570 17414804

[B27] Hickson R. (1980). Interference of strength development by simultaneously training for strength and endurance. Eur. J. Appl. Physiol. Occup. Physiol. 45, 255–263. 10.1007/BF00421333 7193134

[B28] Horne L. Bell G. Fisher B. Warren S. Janowska-Wieczorek A. (1997). Interaction between cortisol and tumour necrosis factor with concurrent resistance and endurance training. Clin. J. Sport Med. Off. J. Can. Acad. Sport Med. 7, 247–251. 10.1097/00042752-199710000-00001 9397322

[B29] Hornum M. Cooper D. M. Brasel J. A. Bueno A. Sietsema K. E. (1997). Exercise-induced changes in circulating growth factors with cyclic variation in plasma estradiol in women. J. Appl. Physiol. 82, 1946–1951. 10.1152/jappl.1997.82.6.1946 9173963

[B30] Hunter G. Demment R. Miller D. (1987). Development of strength and maximum oxygen uptake during simultaneous training for strength and endurance. J. Sports Med. Phys. Fitness 27, 269–275. 3431108

[B31] Ihalainen J. K. Hackney A. C. Taipale R. S. (2019). Changes in inflammation markers after a 10-week high-intensity combined strength and endurance training block in women: the effect of hormonal contraceptive use. J. Sci. Med. Sport 22, 1044–1048. 10.1016/j.jsams.2019.04.002 31186194

[B32] Janse DE Jonge X. A. K. Thompson M. W. Chuter V. H. Silk L. N. Thom J. M. (2012). Exercise performance over the menstrual cycle in temperate and hot, humid conditions. Med. Sci. Sports Exerc. 44, 2190–2198. 10.1249/MSS.0b013e3182656f13 22776870

[B33] Johnston R. E. Quinn T. J. Kertzer R. Vroman N. B. (1997). Strength training in female distance runners: impact on running economy. J. Strength Cond. Res. 11, 224–229. 10.1519/00124278-199711000-00004

[B34] Kanaley J. A. Weltman J. Y. Pieper K. S. Weltman A. Hartman M. L. (2001). Cortisol and growth hormone responses to exercise at different times of day. J. Clin. Endocrinol. Metab. 86, 2881–2889. 10.1210/jc.86.6.2881 11397904

[B35] Kelly C. M. Burnett A. F. Newton M. J. (2008). The effect of strength training on three-kilometer performance in recreational women endurance runners. J. Strength Cond. Res. 22, 396–403. 10.1519/JSC.0b013e318163534a 18550953

[B36] Kirby T. J. Mcbride J. M. Haines T. L. Dayne A. M. (2011). Relative net vertical impulse determines jumping performance. J. Appl. Biomech. Available online at: https://journals.humankinetics.com/view/journals/jab/27/3/article-p207.xml (accessed February 13, 2020). 10.1123/jab.27.3.207 21844609

[B37] Knowles O. E. Aisbett B. Main L. C. Drinkwater E. J. Orellana L. Lamon S. (2019). Resistance training and skeletal muscle protein metabolism in eumenorrheic females: implications for researchers and practitioners. Sports Med. 49, 1637–1650. 10.1007/s40279-019-01132-7 31190324

[B38] Komi P. V. (2000). Stretch-shortening cycle: a powerful model to study normal and fatigued muscle. J. Biomech. 33, 1197–1206. 10.1016/S0021-9290(00)00064-6 10899328

[B39] Kraemer R. R. Heleniak R. J. Tryniecki J. L. Kraemer G. R. Okazaki N. J. Castracane V. D. (1995). Follicular and luteal phase hormonal responses to low-volume resistive exercise. Med. Sci. Sports Exerc. 27, 809–817. 10.1249/00005768-199506000-00004 7658941

[B40] Kraemer W. J. Fleck S. J. Dziados J. E. Harman E. A. Marchitelli L. J. Gordon S. E.. (1993). Changes in hormonal concentrations after different heavy-resistance exercise protocols in women. J. Appl. Physiol. 75, 594–604. 10.1152/jappl.1993.75.2.594 8226457

[B41] Kubo K. Miyamoto M. Tanaka S. Maki A. Tsunoda N. Kanehisa H. (2009). Muscle and tendon properties during menstrual cycle. Int. J. Sports Med. 30, 139–143. 10.1055/s-0028-1104573 19067277

[B42] Kuoppasalmi K. Näveri H. Härkönen M. Adlercreutz H. (1980). Plasma cortisol, androstenedione, testosterone and luteinizing hormone in running exercise of different intensities. Scand. J. Clin. Lab. Invest. 40, 403–409. 10.3109/00365518009101862 7444345

[B43] Laursen P. B. Chiswell S. E. Callaghan J. A. (2005). Should endurance athletes supplement their training program with resistance training to improve performance? Strength Cond. J. 27, 50–55. 10.1519/00126548-200510000-00008

[B44] Lebrun C. M. McKenzie D. C. Prior J. C. Taunton J. E. (1995). Effects of menstrual cycle phase on athletic performance. Med. Sci. Sports Exerc. 27, 437–444. 10.1249/00005768-199503000-00022 7752873

[B45] Leveritt M. Abernethy P. J. Barry B. K. Logan P. A. (1999). Concurrent strength and endurance training. A review. Sports Med. 28, 413–427. 10.2165/00007256-199928060-00004 10623984

[B46] Liguori G. Dwyer G. B. Fitts T. C. Lewis B. (2014). ACSM's Resources for the Health Fitness Specialist, 1st Edn Philadelphia, PA; Baltimore, MD; New York, NY; London; Buenos Aires; Hong Kong; Sydney, NSW; Tokyo: Wolters Kluwer; Lippincott Williams & Wilkins.

[B47] Linnamo V. Pakarinen A. Komi P. V. Kraemer W. J. Häkkinen K. (2005). Acute hormonal responses to submaximal and maximal heavy resistance and explosive exercises in men and women. J. Strength Cond. Res. 19, 566–571. 10.1519/00124278-200508000-00014 16095404

[B48] Mann T. N. Lamberts R. P. Lambert M. I. (2014). High responders and low responders: factors associated with individual variation in response to standardized training. Sports Med. 44, 1113–1124. 10.1007/s40279-014-0197-3 24807838

[B49] McNulty K. L. Elliott-Sale K. J. Dolan E. Swinton P. A. Ansdell P. Goodall S. (2020). The effects of menstrual cycle phase on exercise performance in eumenorrheic women: a systematic review and meta-analysis. Sports Med. 10.1007/s40279-020-01319-3. [Epub ahead of print]. PMC7497427 32661839

[B50] Mikkola J. S. Rusko H. K. Nummela A. T. Paavolainen L. M. Häkkinen K. (2007). Concurrent endurance and explosive type strength training increases activation and fast force production of leg extensor muscles in endurance athletes. J. Strength Cond. Res. 21, 613–620. 10.1519/00124278-200705000-00056 17530970

[B51] Molgat-Seon Y. Peters C. M. Sheel A. W. (2018). Sex-differences in the human respiratory system and their impact on resting pulmonary function and the integrative response to exercise. Curr. Opin. Physiol. 6, 21–27. 10.1016/j.cophys.2018.03.007

[B52] Myllyaho M. M. Ihalainen J. K. Hackney A. C. Valtonen M. Nummela A. Vaara E. (2018). Hormonal contraceptive use does not affect strength, endurance, or body composition adaptations to combined strength and endurance training in women. J. Strength Cond. Res. 10.1519/JSC.0000000000002713. [Epub ahead of print]. 29927884

[B53] Nakamura Y. Aizawa K. Imai T. Kono I. Mesaki N. (2011). Hormonal responses to resistance exercise during different menstrual cycle states. Med. Sci. Sports Exerc. 43, 967–973. 10.1249/MSS.0b013e3182019774 20980927

[B54] Newton R. U. Kraemer W. J. (1994). Developing explosive muscular power: implications for a mixed methods training strategy. Strength Cond. 16, 20–31. 10.1519/1073-6840(1994)016<0020:DEMPIF>2.3.CO;2

[B55] O'Leary C. B. Lehman C. Koltun K. Smith-Ryan A. Hackney A. C. (2013). Response of testosterone to prolonged aerobic exercise during different phases of the menstrual cycle. Eur. J. Appl. Physiol. 113, 2419–2424. 10.1007/s00421-013-2680-1 23812088

[B56] Paavolainen L. Häkkinen K. Hämäläinen I. Nummela A. Rusko H. (1999). Explosive-strength training improves 5-km running time by improving running economy and muscle power. J. Appl. Physiol. 86, 1527–1533. 10.1152/jappl.1999.86.5.1527 10233114

[B57] Petrofsky J. S. LeDonne D. M. Rinehart J. S. Lind A. R. (1976). Isometric strength and endurance during the menstrual cycle. Eur. J. Appl. Physiol. Occup. Physiol. 35, 1–10. 10.1007/BF00444652 1253779

[B58] Pickering C. Kiely J. (2019). Do non-responders to exercise exist—and if so, what should we do about them? Sports Med. 49, 1–7. 10.1007/s40279-018-01041-1 30560423 PMC6349783

[B59] Quadagno D. Faquin L. Lim G. N. Kuminka W. Moffatt R. (1991). The menstrual cycle: does it affect athletic performance? Phys. Sportsmed. 19, 121–124. 10.1080/00913847.1991.11702172

[B60] Reis E. Frick U. Schmidtbleicher D. (1995). Frequency variations of strength training sessions triggered by the phases of the menstrual cycle. Int. J. Sports Med. 16, 545–550. 10.1055/s-2007-973052 8776210

[B61] Roberts B. M. Nuckols G. Krieger J. W. (2020). Sex differences in resistance training. J. Strength Cond. Res. 34, 1448–1460. 10.1519/JSC.0000000000003521 32218059

[B62] Sabag A. Najafi A. Michael S. Esgin T. Halaki M. Hackett D. (2018). The compatibility of concurrent high intensity interval training and resistance training for muscular strength and hypertrophy: a systematic review and meta-analysis. J. Sports Sci. 36, 2472–2483. 10.1080/02640414.2018.1464636 29658408

[B63] Sandbakk Ø. Solli G. S. Holmberg H. C. (2018). Sex differences in world-record performance: the influence of sport discipline and competition duration. Int. J. Sports Physiol. Perform. 13, 2–8. 10.1123/ijspp.2017-0196 28488921

[B64] Sarwar R. Niclos B. B. Rutherford O. M. (1996). Changes in muscle strength, relaxation rate and fatiguability during the human menstrual cycle. J. Physiol. 493, 267–272. 10.1113/jphysiol.1996.sp021381 8735711 PMC1158967

[B65] Schumann M. Küüsmaa M. Newton R. U. Sirparanta A. I. Syväoja H. Häkkinen A.. (2014). Fitness and lean mass increases during combined training independent of loading order. Med. Sci. Sports Exerc. 46, 1758–1768. 10.1249/MSS.0000000000000303 24518195

[B66] Semmler J. G. Enoka R. M. (2000). Neural contributions to the changes in muscle strength, in Biomechanics in Sport: The Scientific Basis of Performance, ed Zaitorsky V. V. M. (oOxford, UK: Blackwell Science Ltd), 3–20. 10.1002/9780470693797.ch1

[B67] Silva R. F. Cadore E. L. Kothe G. Guedes M. Alberton C. L. Pinto S. S.. (2012). Concurrent training with different aerobic exercises. Int. J. Sports Med. 33, 627–634. 10.1055/s-0031-1299698 22562730

[B68] Spurrs R. W. Murphy A. J. Watsford M. L. (2003). The effect of plyometric training on distance running performance. Eur. J. Appl. Physiol. 89, 1–7. 10.1007/s00421-002-0741-y 12627298

[B69] Staron R. S. Hagerman F. C. Hikida R. S. Murray T. F. Hostler D. P. Crill M. T.. (2000). Fiber type composition of the vastus lateralis muscle of young men and women. J. Histochem. Cytochem. 48, 623–629. 10.1177/002215540004800506 10769046

[B70] Støren O. Helgerud J. Støa E. M. Hoff J. Støren Ø. Helgerud J.. (2008). Maximal strength training improves running economy in distance runners. Med. Sci. Sports Exerc. 40, 1087–1092. 10.1249/MSS.0b013e318168da2f 18460997

[B71] Sung E. Han A. Hinrichs T. Vorgerd M. Manchado C. Platen P. (2014). Effects of follicular versus luteal phase-based strength training in young women. Springerplus 3:668. 10.1186/2193-1801-3-668 25485203 PMC4236309

[B72] Taipale R. S. Ihalainen J. K. Jones P. J. Mero A. A. Häkkinen K. Kyröläinen H. (2019). Cold-water immersion combined with active recovery is equally as effective as active recovery during 10 weeks of high-intensity combined strength and endurance training in men. Biomed. Hum. Kinet. 11, 189–192. 10.2478/bhk-2019-0026

[B73] Taipale R. S. Mikkola J. Nummela A. Vesterinen V. Capostagno B. Walker S.. (2010). Strength training in endurance runners. Int. J. Sports Med. 31, 468–476. 10.1055/s-0029-1243639 20432192

[B74] Taipale R. S. Mikkola J. Salo T. Hokka L. Vesterinen V. Kraemer W. J.. (2014). Mixed maximal and explosive strength training in recreational endurance runners. J. Strength Cond. Res. 28, 689–699. 10.1519/JSC.0b013e3182a16d73 23860287

[B75] Tremblay M. S. Copeland J. L. Van Helder W. (2005). Influence of exercise duration on post-exercise steroid hormone responses in trained males. Eur. J. Appl. Physiol. 94, 505–513. 10.1007/s00421-005-1380-x 15942766

[B76] Viru A. Viru M. (2004). Cortisol - essential adaptation hormone in exercise. Int. J. Sports Med. 25, 461–464. 10.1055/s-2004-821068 15346236

[B77] Wikström-Frisén L. Boraxbekk C. J. Henriksson-Larsén K. (2017). Effects on power, strength and lean body mass of menstrual/oral contraceptive cycle based resistance training. J. Sports Med. Phys. Fitness 57, 43–52. 10.23736/S0022-4707.16.05848-5 26558833

[B78] Wilson J. M. Marin J. P. Rhea M. R. Wilson S. M. C. Loenneke J. P. Anderson J. C. (2012). Concurrent training: a meta-analysis examining interference of aerobic and resistance exercises. J. Strength Cond. Res. 26, 2293–2307. 10.1519/JSC.0b013e31823a3e2d 22002517

